# Supplementary material for: Partially Hydrolysed Whey-Based Formulae with Reduced Protein Content Support Adequate Infant Growth and Are Well Tolerated: Results of a Randomised Controlled Trial in Healthy Term Infants
Source: Nutrients. 2019 Jul 19;11(7):1654. doi: 10.3390/nu11071654 (PMC6682927; doi:10.3390/nu11071654)
Supplement: Supplementary file 1 [file nutrients-11-01654-s001.pdf]

Table S1. Infant age and anthropometric data (mean (SD)) at baseline of the PP population.

	pHF1.8 (N = 61)	pHF2.0 (N = 46)	pHF2.27 (N = 48)	Total (N=155)
Age at baseline (days)	7.9 (4.0)	8.1 (4.0)	8.1 (4.3)	8.0 (4.1)
Weight (grams)	3373.6 (427.8)	3398.1 (463.3)	3366.5 (504.2)	3378.7 (460.2)
Length (cm)	50.49 (1.86)	50.75 (2.19)	50.40 (2.44)	50.54 (2.14)
Head circumference of (cm)	35.14 (1.23)	35.38 (1.35)	34.97 (1.52)	35.16 (1.36)

Table S2. Parental characteristics of the PP population.

		pHF1.8 (N = 61)	pHF2.0 (N = 46)	pHF2.27 (N = 48)	Total (N=155)
Height of mother (m)	n; mean (SD)	61; 1.665 (0.065)	46; 1.675 (0.073)	48; 1.659 (0.052)	155; 1.666 (0.064)
Pre-pregnancy weight of mother (kg)	n; mean (SD)	61; 70.86 (17.79)	45; 68.03 (15.32)	48; 66.88 (13.81)	154; 68.79 (15.91)
Smoking of mother during pregnancy	Yes, n (%)	11 (18.0%)	7 (15.2%)	8 (16.7%)	26 (16.8%)
	No, n (%)	50 (82.0%)	39 (84.8%)	40 (83.3%)	129 (83.2%)
Mother diabetes	Yes, n (%)	0	3 (6.5%)	4 (8.3%)	7 (4.5%)
	No, n (%)	61 (100%)	43 (93.5%)	44 (91.7%)	148 (95.5%)
Mother's highest formal education level	Primary school, n (%)	0	1 (2.2%)	1 (2.1%)	2 (1.3%)
	High school, n (%)	39 (63.9%)	29 (63.0%)	29 (60.4%)	97 (62.6%)
	University, n (%)	22 (36.1%)	16 (34.8%)	18 (37.5%)	56 (36.1%)
Height of father (m)	n; mean (SD)	59; 1.791 (0.069)	45; 1.806 (0.067)	47; 1.797 (0.075)	151; 1.798 (0.070)
Weight of father (kg)	n; mean (SD)	59; 81.93 (12.50)	45; 82.40 (14.81)	47; 79.77 (13.65)	151; 81.40 (13.53)

Table S3. Study formula intake (n; Median [Q1-Q3]) by visit of the PP population.

	pHF1.8 (N = 61)	pHF2.0 (N = 46)	pHF2.27 (N = 48)	Total (N = 155)	p-value 1 / p-value 2
**Product intake (mL/kg/day)**					
1 Month	59; 169 (154 - 187)	46; 169 (146 - 192)	47; 180 (160 - 186)	152; 172 (156 - 187)	0.366 / 0.235
2 Month	61; 149 (134 - 161)	46; 150 (138 - 162)	47; 154 (142 - 166)	154; 151 (138 - 163)	0.096 / 0.164
3 Month	60; 137 (127 - 147)	45; 135 (123 - 155)	46; 136 (123 - 150)	151; 136 (125 - 149)	0.945 / 0.671
4 Month	58; 128 (120 - 142)	44; 132 (120 - 145)	46; 126 (118 - 139)	148; 129 (119 - 142)	0.726 / 0.334
**Product intake (mL/day), by visit**					
1 Month	59; 703 (613 - 768)	46; 707 (626 - 766)	47; 764 (653 - 817)	152; 711 (636 - 790)	0.103 / 0.027
2 Month	61; 738 (673 - 834)	46; 764 (686 - 812)	47; 821 (757 - 890)	154; 768 (689 - 849)	0.014 / 0.005
3 Month	60; 792 (728 - 874)	45; 786 (690 - 871)	46; 820 (747 - 935)	151; 797 (714 - 886)	0.431 / 0.134
4 Month	58; 830 (749 - 927)	44; 847 (749 - 945)	46; 839 (759 - 968)	148; 840 (750 - 945)	0.714 / 0.891
p-value 1: Comparison pHF1.8 vs. pHF2.27, p-value 2: Comparison pHF2.0 vs. pHF2.27, Van Elteren test					

Table S4. Z-scores (n; mean [95% CI]) of growth outcome parameters of the PP population.

	pHF1.8 (N = 61)	pHF2.0 (N = 46)	pHF2.27 (N = 48)	Total (N = 155)
**Weight-for-age z-score (WAZ)**				
Birth	61; 0.06 (-0.17; 0.29)	46; 0.09 (-0.16; 0.34)	48; 0.03 (-0.24; 0.30)	155; 0.06 (-0.08; 0.20)
Baseline	61; -0.20 (-0.42;0.01)	46; -0.16 (-0.39;0.07)	48; -0.26 (-0.53;0.00)	155; -0.21 (-0.34;-0.07)
1 Month	61; -0.20 (-0.44;0.04)	46; -0.19 (-0.39;0.02)	48; -0.17 (-0.40;0.06)	155; -0.19 (-0.32;-0.06)
2 Month	61; -0.27 (-0.51;-0.02)	46; -0.27 (-0.47;-0.07)	48; -0.21 (-0.44;0.03)	155; -0.25 (-0.38;-0.12)
3 Month	61; -0.23 (-0.49;0.04)	46; -0.35 (-0.56;-0.15)	48; -0.12 (-0.36;0.13)	155; -0.23 (-0.37;-0.09)
4 Month	61; -0.15 (-0.42;0.11)	46; -0.29 (-0.51;-0.06)	48; -0.01 (-0.25;0.24)	155; -0.15 (-0.29;-0.00)
12 Month	60; -0.01 (-0.31;0.30)	43; 0.02 (-0.26;0.29)	46; 0.08 (-0.15;0.31)	149; 0.03 (-0.13;0.18)
**Length-for-age z-score (LAZ)**				
Birth	61; -0.04 (-0.31;0.23)	46; 0.33 (0.00;0.65))	48, -0.04 (-0.37;0.29)	155; 0.07 (-0.10;0.24)
Baseline	60; -0.22 (-0.47;0.03)	46; -0.08 (-0.38;0.22)	48; -0.30 (-0.63;0.02)	154; -0.20 (-0.37;-0.04)

	pHF1.8 (N = 61)	pHF2.0 (N = 46)	pHF2.27 (N = 48)	Total (N = 155)
1 Month	61; -0.41 (-0.68;-0.15)	46; -0.06 (-0.34;0.22)	48; -0.42 (-0.72;-0.12)	155; -0.31 (-0.47;-0.15)
2 Month	61; -0.38 (-0.65;-0.10)	46; 0.02 (-0.27;0.32)	48; -0.24 (-0.55;0.08)	155; -0.21 (-0.38;-0.05)
3 Month	61; -0.34 (-0.63;-0.06)	46; 0.04 (-0.25;0.33)	48; -0.22 (-0.53;0.09)	155; -0.19 (-0.36;-0.02)
4 Month	61; -0.04 (-0.34;0.26)	46; 0.24 (-0.02;0.49)	48; 0.03 (-0.27;0.33)	155; 0.07 (-0.10;0.23)
12 Month	60; -0.06 (-0.40;0.29)	43; 0.16 (-0.17;0.48)	46; -0.05 (-0.38;0.28)	149; 0.01 (-0.19;0.20)
**Weight-for-Length z-score (LAZ)**				
Birth	60; 0.22 (-0.04;0.49)	46; -0.16 (-0.40;0.07)	48; 0.19 (-0.10;0.48)	154; 0.10 (-0.06;0.25)
Baseline	60; -0.31 (-0.50;-0.12)	46; -0.45 (-0.70;-0.20)	48; -0.33 (-0.62;-0.04)	154; -0.36 (-0.49;-0.22)
1 Month	61; 0.19 (-0.06;0.45)	46; -0.26 (-0.56;0.03)	48; 0.25 (-0.02;0.53)	155; 0.08 (-0.08;0.23)
2 Month	61; 0.19 (-0.04;0.43)	46; -0.32 (-0.62;-0.03)	48; 0.11 (-0.16;0.38)	155; 0.01 (-0.14;0.17)
3 Month	61; 0.14 (-0.09;0.37)	46; -0.47 (-0.71;-0.22)	48; 0.17 (-0.07;0.40)	155; -0.03 (-0.17;0.11)
4 Month	61; -0.09 (-0.33;0.14)	46; -0.56 (-0.81;-0.32)	48; 0.03 (-0.20;0.27)	155; -0.19 (-0.33;-0.05)
12 Month	60; 0.05 (-0.21;0.30)	43; -0.06 (-0.35;0.23)	46; 0.15 (-0.11;0.41)	149; 0.05 (-0.10;0.20)
**Head Circumference-for-age z-score (HCAZ)**				
Birth	61, 0.05 (-0.25; 0.33)	46; 0.22 (-0.15;0.59)	48; -0.10 (-0.39;0.20)	155; 0.05 (-0.13;0.23)
Baseline	60; 0.16 (-0.09;0.40)	46; 0.36 (0.07;0.65)	48; -0.03 (-0.35;0.29)	154; 0.16 (-0.00;0.32)
1 Month	61; 0.33 (0.08;0.58)	46; 0.56 (0.31;0.81)	48; 0.33 (0.05;0.62)	155; 0.40 (0.25;0.55)
2 Month	61; 0.44 (0.20;0.68)	46; 0.47 (0.24;0.70)	48; 0.38 (0.12;0.63)	155; 0.43 (0.29;0.57)
3 Month	61; 0.49 (0.23;0.75)	46; 0.47 (0.26;0.68)	48; 0.43 (0.21;0.66)	155; 0.47 (0.33;0.60)
4 Month	61; 0.48 (0.22;0.74)	46; 0.51 (0.28;0.75)	48; 0.53 (0.32;0.75)	155; 0.51 (0.37;0.65)
12 Month	60; 0.58 (0.30;0.86)	42; 0.46 (0.14;0.79)	45; 0.58 (0.36;0.81)	147; 0.55 (0.39;0.71)

Table S5. Severity of GI Symptoms (Median [Min. ; Max.]) of the ITT population.

	pHF1.8 (N = 72)	pHF2.0 (N = 67)	pHF2.27 (N = 688)	Total (N = 207)	p-value 1 / p-value 2
Vomiting					
1 Month	0.00 (0.0 ; 1.9)	0.00 (0.0 ; 2.7)	0.00 (0.0 ; 1.2)	0.00 (0.0 ; 2.7)	0.222 / 0.701
2 Month	0.00 (0.0 ; 2.6)	0.00 (0.0 ; 2.8)	0.00 (0.0 ; 2.9)	0.00 (0.0 ; 2.9)	0.256 / 0.129
3 Month	0.00 (0.0 ; 2.0)	0.00 (0.0 ; 1.7)	0.00 (0.0 ; 1.9)	0.00 (0.0 ; 2.0)	0.126 / 0.059
4 Month	0.00 (0.0 ; 2.3)	0.00 (0.0 ; 1.7)	0.00 (0.0 ; 1.4)	0.00 (0.0 ; 2.3)	0.804 / 0.163
Burping					
1 Month	1.00 (0.0 ; 2.1)	1.00 (0.0 ; 2.0)	0.71 (0.0 ; 2.0)	1.00 (0.0 ; 2.1)	0.218 / 0.369
2 Month	1.00 (0.0 ; 2.4)	1.00 (0.0 ; 2.2)	1.00 (0.0 ; 2.0)	1.00 (0.0 ; 2.4)	0.555 / 0.815
3 Month	0.64 (0.0 ; 1.9)	0.71 (0.0 ; 2.0)	0.86 (0.0 ; 2.1)	0.71 (0.0 ; 2.1)	0.606 / 0.807
4 Month	0.71 (0.0 ; 2.9)	0.29 (0.0 ; 1.7)	0.36 (0.0 ; 2.0)	0.43 (0.0 ; 2.9)	0.961 / 0.977
Flatulence					
1 Month	1.14 (0.0 ; 3.0)	1.29 (0.0 ; 2.9)	1.29 (0.0 ; 2.7)	1.20 (0.0 ; 3.0)	0.287 / 0.562
2 Month	1.00 (0.0 ; 2.4)	1.29 (0.0 ; 3.0)	1.14 (0.0 ; 3.0)	1.14 (0.0 ; 3.0)	0.195 / 0.873
3 Month	0.86 (0.0 ; 2.0)	1.00 (0.0 ; 2.1)	0.86 (0.0 ; 2.3)	1.00 (0.0 ; 2.3)	0.701 / 0.270
4 Month	0.57 (0.0 ; 2.1)	1.00 (0.0 ; 2.6)	0.86 (0.0 ; 2.1)	0.86 (0.0 ; 2.6)	0.453 / 0.321
Diarrhoea					
1 Month	0.00 (0.0 ; 1.7)	0.00 (0.0 ; 1.9)	0.00 (0.0 ; 1.6)	0.00 (0.0 ; 1.9)	0.166 / 0.227
2 Month	0.00 (0.0 ; 2.9)	0.00 (0.0 ; 2.3)	0.00 (0.0 ; 2.0)	0.00 (0.0 ; 2.9)	0.120 / 0.750
3 Month	0.00 (0.0 ; 1.9)	0.00 (0.0 ; 1.3)	0.00 (0.0 ; 2.1)	0.00 (0.0 ; 2.1)	0.027 / 0.695
4 Month	0.00 (0.0 ; 1.7)	0.00 (0.0 ; 1.4)	0.00 (0.0 ; 2.0)	0.00 (0.0 ; 2.0)	0.057 / 0.123
Constipation					
1 Month	0.00 (0.0 ; 1.3)	0.00 (0.0 ; 0.6)	0.00 (0.0 ; 1.0)	0.00 (0.0 ; 1.3)	0.442 / 0.964
2 Month	0.00 (0.0 ; 0.6)	0.00 (0.0 ; 0.4)	0.00 (0.0 ; 0.7)	0.00 (0.0 ; 0.7)	0.591 / 0.881
3 Month	0.00 (0.0 ; 0.4)	0.00 (0.0 ; 0.2)	0.00 (0.0 ; 0.1)	0.00 (0.0 ; 0.4)	0.650 / 0.961
4 Month	0.00 (0.0 ; 0.1)	0.00 (0.0 ; 0.0)	0.00 (0.0 ; 0.0)	0.00 (0.0 ; 0.1)	0.403 / -
Diaper dermatitis					
1 Month	0.00 (0.0 ; 1.1)	0.00 (0.0 ; 1.3)	0.00 (0.0 ; 3.0)	0.00 (0.0 ; 3.0)	0.785 / 0.106
2 Month	0.00 (0.0 ; 3.0)	0.00 (0.0 ; 2.0)	0.00 (0.0 ; 1.3)	0.00 (0.0 ; 3.0)	0.859 / 0.899
3 Month	0.00 (0.0 ; 1.6)	0.00 (0.0 ; 1.0)	0.00 (0.0 ; 1.1)	0.00 (0.0 ; 1.6)	0.088 / 0.872
4 Month	0.00 (0.0 ; 1.3)	0.29 (0.0 ; 1.0)	0.14 (0.0 ; 3.0)	0.14 (0.0 ; 3.0)	0.020 / 0.095
Colic (cramps)					
1 Month	0.86 (0.0 ; 2.3)	1.00 (0.0 ; 2.4)	1.00 (0.0 ; 2.4)	1.00 (0.0 ; 2.4)	0.282 / 0.646
2 Month	0.71 (0.0 ; 2.7)	0.71 (0.0 ; 2.6)	0.86 (0.0 ; 2.9)	0.71 (0.0 ; 2.9)	0.129 / 0.630
3 Month	0.00 (0.0 ; 1.9)	0.29 (0.0 ; 2.0)	0.14 (0.0 ; 2.6)	0.14 (0.0 ; 2.6)	0.284 / 0.730
4 Month	0.00 (0.0 ; 1.7)	0.29 (0.0 ; 2.7)	0.14 (0.0 ; 1.6)	0.14 (0.0 ; 2.7)	0.085 / 0.708
Regurgitation					

	pHF1.8 (N = 72)	pHF2.0 (N = 67)	pHF2.27 (N = 688)	Total (N = 207)	p-value 1 / p-value 2
1 Month	0.71 (0.0 ; 2.6)	0.71 (0.0 ; 2.4)	0.83 (0.0 ; 2.0)	0.71 (0.0 ; 2.6)	0.446 / 0.458
2 Month	0.57 (0.0 ; 2.6)	0.14 (0.0 ; 2.3)	0.57 (0.0 ; 2.7)	0.43 (0.0 ; 2.7)	0.744 / 0.367
3 Month	0.29 (0.0 ; 2.0)	0.29 (0.0 ; 1.9)	0.57 (0.0 ; 2.6)	0.43 (0.0 ; 2.6)	0.242 / 0.296
4 Month	0.43 (0.0 ; 2.4)	0.14 (0.0 ; 2.3)	0.71 (0.0 ; 2.0)	0.43 (0.0 ; 2.4)	0.299 / 0.089

Van Elteren test, p-value 1: Comparison pHF1.8 vs. pHF2.27, p-value 2: Comparison pHF2.0 vs. pHF2.27
